# Perioperative Carcinoid Crisis: A Systematic Review and Meta-Analysis

**DOI:** 10.3390/cancers14122966

**Published:** 2022-06-16

**Authors:** Aileen Xu, Pilar Suz, Tea Reljic, Abhirup C. Are, Ambuj Kumar, Benjamin Powers, Jonathan Strosberg, Jason W. Denbo, Jason B. Fleming, Daniel A. Anaya

**Affiliations:** 1 Department of Gastrointestinal Oncology, H. Lee Moffitt Cancer Center & Research Institute, Tampa, FL 33612, USA; aileenx@usf.edu (A.X.); acare2019@hotmail.com (A.C.A.); benjamin.powers@moffitt.org (B.P.); jonathan.strosberg@moffitt.org (J.S.); jason.denbo@moffitt.org (J.W.D.); jason.fleming@moffitt.org (J.B.F.); 2 USF Health Morsani College of Medicine, University of South Florida, Tampa, FL 33620, USA; 3 Department of Anesthesiology, H. Lee Moffitt Cancer Center & Research Institute, Tampa, FL 33612, USA; maria.suzruiz@moffitt.org; 4 Research Methodology and Biostatistics Core, Office of Research, USF Health Morsani College of Medicine, University of South Florida, Tampa, FL 33620, USA; treljic@usf.edu (T.R.); akumar6@usf.edu (A.K.)

**Keywords:** neuroendocrine tumor, carcinoid crisis, prophylactic octreotide, meta-analysis

## Abstract

**Simple Summary:**

Intraoperative carcinoid crisis (CC) is thought to be a potentially lethal complication for patients with neuroendocrine tumors (NET). Though perioperative octreotide is often recommended for prevention, recent NET society guidelines raised concerns regarding limited data supporting the use of perioperative octreotide to prevent CC. The aim of our meta-analysis was to evaluate the existing evidence characterizing CC and the efficacy of prophylactic octreotide. We found that CC occurs frequently in patients having midgut NETs surgery, specifically those with NET liver metastasis, and is associated with worse postoperative outcomes. Our findings did not show a decreased risk in CC with prophylactic octreotide and questioned the advantage of routine prophylactic octreotide.

**Abstract:**

Background: Surgery is the only curative option for patients with neuroendocrine tumors (NET) and is also indicated for debulking of liver metastasis. Intraoperative carcinoid crisis (CC) is thought to be a potentially lethal complication. Though perioperative octreotide is often recommended for prevention, recent NET society guidelines raised concerns regarding limited data supporting its use. We sought to evaluate existing evidence characterizing CC and evaluating the efficacy of prophylactic octreotide. Methods: A systematic review was performed on studies including patients having surgery for well-differentiated NET and/or NET liver metastasis (2000–2021), and reporting data on the incidence, risk factors, or prognosis of CC, and/or use of prophylactic octreotide. Meta-analysis was performed using random-effects models. Results: Eight studies met inclusion criteria (*n* = 943 operations). The pooled incidence of CC was 19% (95% CI [0.06–0.36]). Liver metastasis (odds ratio 2.85 [1.49–5.47]) and gender (male 0.58 [0.34–0.99]) were the only significant risk factors. The occurrence of CC was associated with increased risk of major postoperative complications (2.12 [1.03–4.35]). The use of prophylactic octreotide was not associated with decreased risk of CC (0.73 [0.32–1.66]). Notably, there was no standard prophylactic octreotide strategy used. Conclusions: Intraoperative carcinoid crisis is a common complication occurring in up to 20% of patients with midgut NET and/or liver metastasis undergoing surgery. Prophylactic octreotide may not provide an efficient way to prevent this complication. Future studies should focus on prospective evaluation of well-defined prophylactic protocols using a standardized definition for CC.

## 1. Introduction

Neuroendocrine tumors (NET) encompass a heterogenous but histologically defined tumor type that can originate from different organs, most commonly the small bowel, lungs, and rectum [1,2]. In the US, the estimated incidence of NETs was 6.98 per 100,000 in 2012, a six-fold increase from 1973 [3]. Similarly, the 20-year limited-duration prevalence of NETs was estimated to be 171,321 in 2014, a significant increase from the estimate of 103,312 in 2004 [3]. For well-differentiated NETs with locoregional and/or resectable distant disease, surgery is the only potentially curative treatment [4,5,6,7]. Debulking surgery for unresectable liver metastases is also indicated for symptom management and improves outcomes (overall survival) for a selected group of patients [8,9]. Patients with gastroenteropancreatic (GEP) NETs (in particular those with midgut NET, i.e., small bowel), and/or those with metastatic disease to the liver, are at risk of intraoperative carcinoid crisis at time of surgery, which is characterized by sudden onset of hemodynamic instability that can be accompanied by other findings such as flushing or bronchospasms [10]. Intraoperative carcinoid crisis is a potentially lethal event that has been associated with postoperative complications [2]. Despite the serious impact of carcinoid crisis, the reported incidence ranges significantly, and data on risk factors, prevention, and prognosis varies across studies, with many aspects of its presentation and management still undefined and driven by dogma [11].

A common hypothesis about the cause of carcinoid crisis is that it has a similar pathophysiology as carcinoid syndrome, which is thought to be related to the release of large amounts of vasoactive compounds including serotonin, histamine, and bradykinin, among others [12,13]. This results in a constellation of symptoms including flushing, diarrhea, bronchospasms, and hemodynamic instability [14]. The predominant strategy for carcinoid crisis prevention has therefore been the use of perioperative octreotide, a somatostatin receptor analog used to treat carcinoid syndrome [15,16]. However, there is no standard scheme for the dose, timing, or administration method of octreotide to prevent carcinoid crisis. Preventative strategies vary widely and range from a single preoperative dose of short-acting octreotide to more complex strategies that span the perioperative period and can even include long-acting somatostatin analogues (SSAs). Further, at this point it is unclear if prophylactic octreotide is effective in preventing carcinoid crisis [2,10] and recent NET society guidelines, including the 2017 European Neuroendocrine Tumor Society (ENETS) guidelines and the 2020 North American Neuroendocrine Tumor Society (NANETS) guidelines, have both drawn attention to the paucity of data surrounding the effectiveness of prophylactic octreotide, and the inconsistent use of the term carcinoid crisis, emphasizing the need for higher-level data before prophylactic octreotide can be recommended [10,17].

Given the above considerations, the objective of the current meta-analysis was to systematically evaluate the available data on intraoperative carcinoid crisis from studies in adult patients with midgut NETs and/or NET liver metastases, having an operation. Our goal was to examine available data and characterize different aspects of intraoperative carcinoid crisis including incidence, risk factors, and prognosis, while focusing primarily on the available data examining effectiveness of octreotide on preventing carcinoid crisis. Our working hypothesis is that prophylactic octreotide does not prevent the occurrence of intraoperative carcinoid crisis.

## 2. Materials and Methods

### 2.1. Data Sources

**Figure A1:**
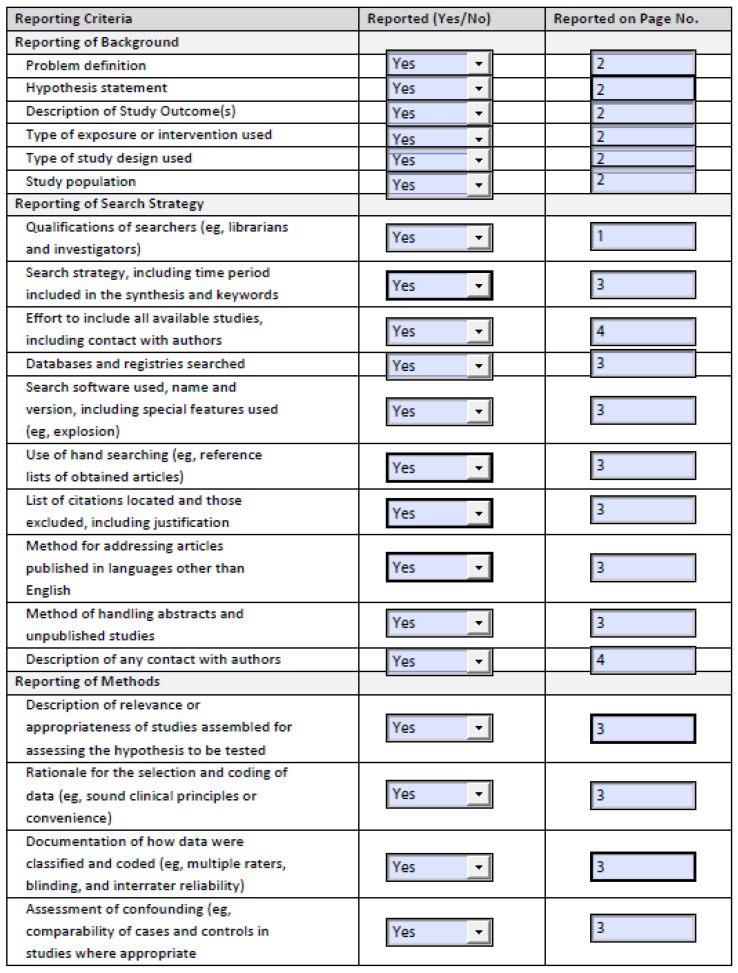

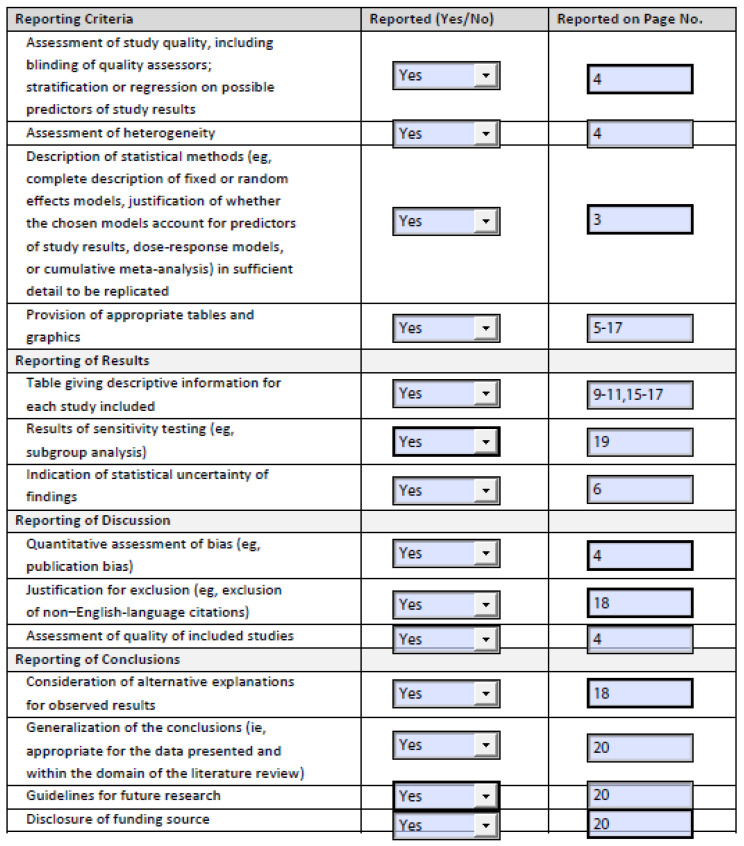
MOOSE (Meta-Analysis of Observational Studies in Epidemiology) checklist.

We performed an electronic search of PubMed, Embase, and Cochrane Library on 30 June 2021. For Embase the search terms were: (neuroendocrine AND tumor OR (carcinoid AND tumor) OR carcinoids) AND (surgery OR operation) AND (hemodynamic AND instability OR (intraoperative AND carcinoid AND syndrome) OR (carcinoid AND crisis) OR (intraoperative AND hypotension) OR (intraoperative AND hypertension) NOT pheochromocytoma in All Fields. For PubMed the search terms were: (neuroendocrine tumor OR carcinoid tumor OR carcinoids) AND (surgery OR operation) AND (hemodynamic instability OR intraoperative carcinoid syndrome OR carcinoid crisis OR intraoperative hypotension OR intraoperative hypertension) NOT Pheochromocytoma. For Cochrane Library, the search terms were: neuroendocrine tumor OR carcinoid tumor OR carcinoids in All Text AND surgery OR operation in All Text AND hemodynamic instability OR intraoperative carcinoid syndrome OR carcinoid crisis OR intraoperative hypotension OR intraoperative hypertension in All Text NOT pheochromocytoma in Keyword. A filter for records from 1 January 2000 through 30 June 2021 was applied. We compiled the search results from the three databases and discarded duplicates. Additionally, we hand-searched the reference lists of the North American Neuroendocrine Tumor Society (NANETS) and the European Neuroendocrine Tumor Society (ENETS) guidelines from 2015 or later, for related manuscripts. This systematic review and meta-analysis follow established PRISMA and MOOSE guidelines (see Figure A1) and were registered in the PROSPERO database (CRD42022330309).

### 2.2. Study Selection Criteria

Two reviewers (AX and PS) independently reviewed the titles and abstracts of unique references for study relevance and then performed the full text review to identify included studies. Discrepancies were resolved by consensus with a third reviewer (DAA). References were included in the systematic review if they described a randomized or nonrandomized study which enrolled adults (age 18 or older) with low-to-intermediate grade neuroendocrine tumors (NETs) of the midgut, and/or patients with NET liver metastasis, and evaluated the incidence, risk factors, prevention, or outcomes of carcinoid crisis.

References were excluded if they were (1) not a study in humans, (2) were not published in English, (3) did not report findings from a primary study (i.e., a systematic review, meta-analysis, review, editorial/letter, case report, description of study protocol, or guidelines), or (4) enrolled less than 40 participants. For studies that had multiple publications, the most up-to-date and complete publication was kept.

### 2.3. Data Extraction and Tabulation

Two reviewers (AX and PS) independently performed the data extraction using a standardized data extraction form. Any disagreement between the two reviewers was resolved by a third reviewer (DAA). Data extracted from studies included general study characteristics (e.g., authors, institution type, study design, and enrollment period), study population characteristics (e.g., tumor types and stages, carcinoid syndrome, carcinoid heart disease, primary tumor location, and surgery types), definition of carcinoid crisis, incidence of carcinoid crisis, rate of prophylaxis use (i.e., administration and dosage of octreotide), and risk factors (e.g., age, gender, primary tumor location, carcinoid syndrome, carcinoid heart disease, and presence of liver metastases). When available, we extracted data on the total number of individuals evaluated and total number with event. When counts were not provided, we used percentages reported in the study and total number evaluated to estimate the number of events.

### 2.4. Risk of Bias/Assessment of Confounding

The Newcastle–Ottawa Quality Assessment Scale for assessment of risk of bias in case–control studies was used to assess the risk of bias in the eligible studies [18].

### 2.5. Data Analysis and Statistical Methods

The proportion of patients who developed carcinoid crisis was computed for each study. Proportions from individual studies were pooled under the random-effects model. The proportion and 95% confidence interval (CI) are reported. Furthermore, to evaluate the association between use of prophylaxis and other risk factors and the incidence of carcinoid crisis, odds ratios were computed for each study with available data. When data were available from studies with similar populations, and methods of measurement for exposures and outcomes, they were included in a meta-analysis. Odds ratios from individual studies were pooled under the random-effects model and the pooled odds ratios, 95% CIs, and p-values are reported. Heterogeneity of pooled studies was evaluated using the I2 statistic. An I2 < 30% was considered low heterogeneity, I2 < 60% moderate heterogeneity, and I2 ≥ 60% high heterogeneity. To explore possible sources of heterogeneity of pooled studies and to test the robustness of the results, we performed a subgroup analysis according to the definition of carcinoid crisis used by the authors. Additionally, we performed a sensitivity analysis according to the timing of prophylaxis treatment. All meta-analyses were conducted using STATA 16 [19]. This work is reported according to the Meta-Analysis of Observational Studies in Epidemiology (MOOSE) guidelines; see MOOSE checklist in Appendix A Figure A1.

## 3. Results

### 3.1. Search Result

**Figure A2:**
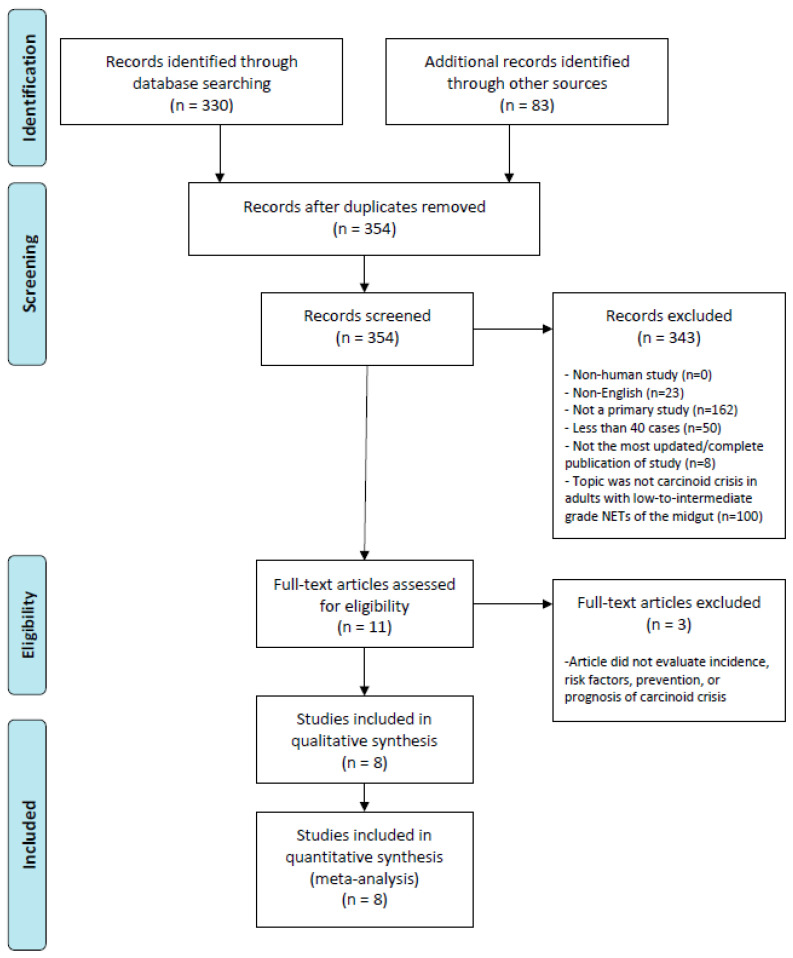
PRISMA flow diagram for study selection.

Our search identified 354 references. The study selection process is reported in Appendix A Figure A2. Two records passed abstract review but were conference abstracts. Authors were contacted and did not have full texts, so the two records were excluded. Eight studies which enrolled 864 patients (943 operations) met all inclusion criteria [20,21,22,23,24,25,26,27].

### 3.2. Study and Patient Characteristics

The patient enrollment period from the studies included ranged from 1983 to 2017, with a total of 864 unique patients and 943 operations included. Three studies reported on patients having more than one operation [22,23,25]. Though all studies examined surgical patients with NETs, other study population characteristics varied (Table 1). All studies included patients with neuroendocrine tumors of gastrointestinal origin (midgut), with 1 of the 8 studies also including patients with history of lung NET as the primary site and now presenting with liver metastasis [26]. In 5 studies, all patients had metastatic disease, with 4 studies selecting patients with liver metastasis (with or without other sites) [20,25,26,27], while the 5th study included patients with metastasis not exclusive of the liver (i.e., metastasis could be at other sites and not include the liver) [23]. All studies included patients with carcinoid syndrome, with 6 studies reporting the proportion of patients presenting with carcinoid syndrome, ranging between 46.7 and 85.2% [21,22,23,24,26,27]. One study excluded patients with carcinoid heart disease [26], and another did not specify whether carcinoid heart disease patients were included [23]. Otherwise, carcinoid heart disease was present in 2.1–20.2% of patients in the remaining studies [20,21,22,24,25,27]. One study did not have detailed information regarding the specific operative procedure/s performed [23]. For the other 7 studies, surgical procedures were all intra-abdominal, gastrointestinal operations with the extent ranging from a simple cholecystectomy to primary tumor resection (e.g., small bowel/mesenteric resection) or hepatectomy, and in some cases including patients having debulking single- and multi-organ procedures [20,21,22,24,25,26,27].

### 3.3. Risk of Bias of Studies Included

All studies were single-center; 6 were retrospective [20,21,23,24,25,27] and 2 were prospective studies [22,26]. Since all studies reported outcomes according to carcinoid crisis status, we classified all studies as case–control. The overall risk of bias of the 8 studies included is summarized in Appendix A Table A1 (Newcastle–Ottawa Quality Assessment Scale—risk of bias assessment). Briefly, the case status (carcinoid crisis) was clearly defined in 87.5% (7/8) of studies. Fifty percent (4/8) of the studies included all consecutive patients with carcinoid crisis and thus were judged to have a representative sample of cases. It is not clear if the remaining 4 studies enrolled all consecutive patients. The controls (i.e., individuals without carcinoid crisis) were recruited from the same population as the cases in all studies and the definition of controls was clearly stated in 87.5% (7/8) of studies. Seventy-five percent (6/8) of the studies ensured that the cases and controls were comparable to each other through matching or adjustment. The ascertainment of exposure was completed using secure records for both cases and controls in all studies and thus was judged to be at low risk of bias for all studies. Most patients in all studies had complete data, so risk of non-response/attrition bias is low across all studies.

### 3.4. Outcomes

All studies included hemodynamic changes as the primary finding supporting the diagnosis of carcinoid crisis (Table 1); in 7 studies, a systolic blood pressure (SBP) <80 mmHg, was used as the threshold for diagnosis, while in the eighth study the definition was based on a change in SBP representing a drop ≥40% [24]. In all but one study, it was an explicit pre-requisite to rule out other causes of hemodynamic instability to confirm carcinoid crisis as the predisposing event [20]. In 5 studies, a time-dependent effect was necessary to define carcinoid crisis, only if the findings lasted for 10 min or longer [20,21,23,25,27] while for 2 studies no time limit was used [22,26]. The eighth study used 4 different levels of certainty for diagnosing carcinoid crisis ranging from physiologic changes treated with octreotide to life-threatening changes refractory to treatment, and no time-limit was included [24]. In all 8 studies, other physiologic changes were also included as alternatives for diagnosis of carcinoid crisis including tachycardia (HR > 120/min), hypertension (>180 mmHg), bronchospasm, flushing, urticaria, and acidosis [20,21,22,23,24,25,26,27]. Lastly, in 3 studies, carcinoid crisis could also be declared if the physiologic changes during surgery were considered due to carcinoid crisis by the treating surgeon and/or anesthesiologist [21,23,27], while in 2 other studies this was a pre-requisite to define carcinoid crisis occurrence [22,26].

Two studies had narrow definitions for carcinoid crisis but included broader criteria when defining the main study outcome [24,27]. This analysis used the broadest definitions from these 2 studies as carcinoid crisis because they were more comparable to the definition of carcinoid crisis used across the other studies. In the context of these differences, the incidence of carcinoid crisis when using the definitions presented in each study ranged from 0 to 35%. When considering carcinoid crisis occurrence based on hemodynamic changes for all studies, the range was 0–56%.

#### 3.4.1. Incidence of Carcinoid Crisis

**Figure 1:**
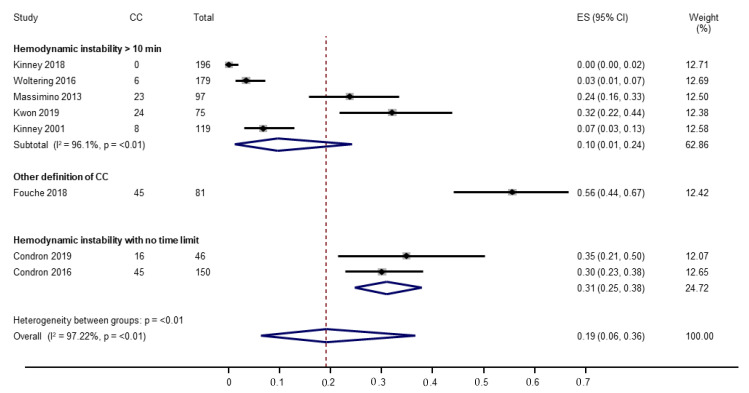
Forest plot evaluating incidence of carcinoid crisis [20,21,22,23,24,25,26,27].

The pooled incidence of carcinoid crisis (8 studies, 943 surgeries) was 19% (95% CI, 6–36%). The level of heterogeneity was high (I2 = 97.2%). The pooled incidence of carcinoid crisis in studies using hemodynamic instability of at least 10 min as part of the definition (5 studies, 666 surgeries) was 10% (95% CI, 1–24%). The pooled incidence of carcinoid crisis in studies using no time limit as part of the carcinoid crisis definition (2 studies, 196 surgeries) was 31% (95% CI, 25–38%). The incidence of carcinoid crisis in the single study (81 surgeries) which used 4 levels of certainty of intraoperative carcinoid syndrome was 56% (95% CI, 44–67%). (Figure 1). The test of interaction between the definition subgroups was significant (*p* < 0.001).

Sensitivity analysis was conducted by excluding a study that included patients with lung primary NETs [26], which resulted in a pooled incidence of 17% (95% CI, 5–36%).

#### 3.4.2. Risk Factors for Carcinoid Crisis

Six studies evaluated risk factors associated with carcinoid crisis, which is summarized in Table 2.

**Figure 2:**
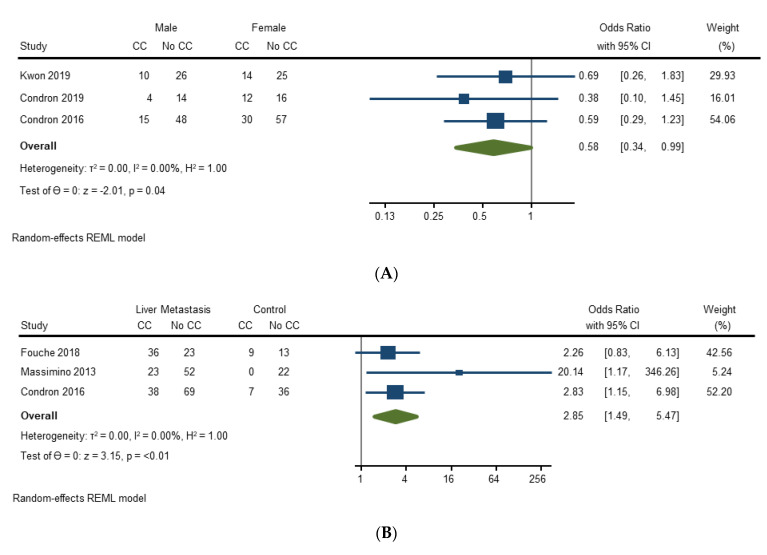

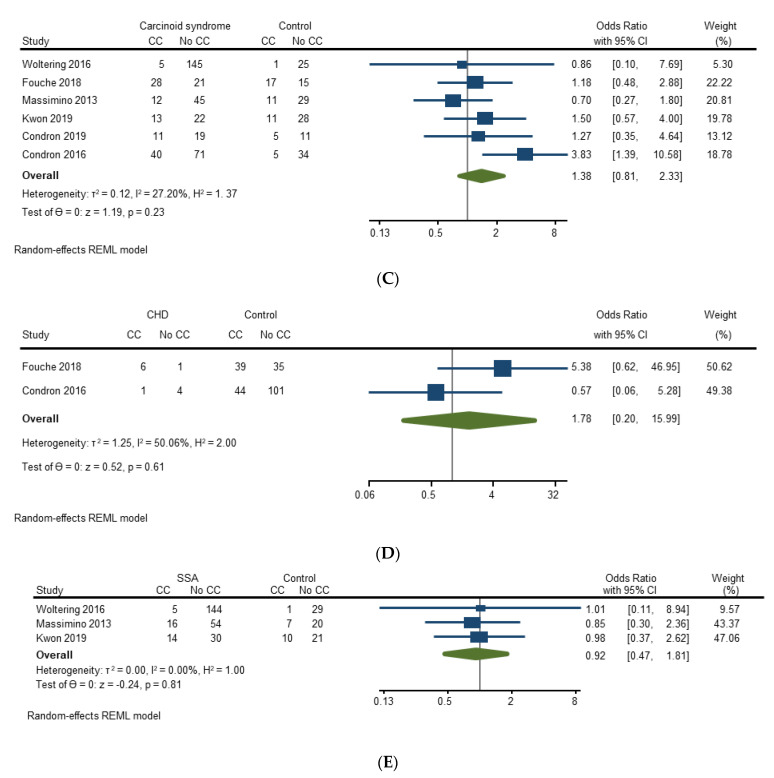
(**A**) Forest plot evaluating risk factors—gender; (**B**) Forest plot evaluating risk factors—liver metastasis; (**C**) Forest plot evaluating risk factors—carcinoid syndrome; (**D**) Forest plot evaluating risk factors—carcinoid heart disease (CHD); (**E**) Forest plot evaluating risk factors—long-acting somatostatin analogues [21,22,23,24,26,27].

Three studies (271 surgeries) assessed the effect of gender on development of carcinoid crisis. Male gender compared to female gender was associated with lower pooled odds of developing carcinoid crisis in 3 studies [OR = 0.58 (95% CI, 0.34–0.99); *p* = 0.04]. There was no heterogeneity between studies (I2 = 0%) (Figure 2A). Sensitivity analysis was conducted by excluding a study that included patients with lung primary NETs [26], which resulted in gender no longer having a significant association with carcinoid crisis [OR 0.63 (95% CI, 0.35–1.12); *p* = 0.12].

Three studies (328 surgeries) assessed the effect of liver metastasis on development of carcinoid crisis. Liver metastasis compared to no liver metastasis was associated with higher pooled odds of developing carcinoid crisis [OR 2.85 (95% CI, 1.49–5.47); *p* ≤ 0.01]. There was no heterogeneity between studies (I2 = 0%) (Figure 2B).

Six studies (624 surgeries) assessed the effect of carcinoid syndrome on development of carcinoid crisis. Carcinoid syndrome compared to no carcinoid syndrome was not associated with higher pooled odds of developing carcinoid crisis [OR = 1.38 (95% CI, 0.81–2.33); *p* = 0.23]. The heterogeneity between studies was low (I2 = 27.2%) (Figure 2C). Sensitivity analysis was conducted by excluding a study that included patients with lung primary NETs [26], which showed that carcinoid syndrome remained not significantly associated with developing carcinoid crisis [OR 1.39 (95% CI, 0.75–2.59); *p* = 0.29].

Two studies (231 surgeries) assessed the effect of carcinoid heart disease on development of carcinoid crisis. Carcinoid heart disease compared to no carcinoid heart disease was not associated with higher pooled odds of developing carcinoid crisis [OR 1.78 (95% CI, 0.20–15.99); *p* = 0.61]. The heterogeneity between studies was moderate (I2 = 50.1%) (Figure 2D).

Three studies (351 surgeries) assessed the effect of long-acting somatostatin synthetic analogs (SSA) use on development of carcinoid crisis. Long-acting SSA use compared to no long-acting SSA use was not associated with higher pooled odds of developing carcinoid crisis [OR 0.92 (95% CI, 0.47–1.81); *p* = 0.81]. There was no heterogeneity between studies (I2 = 0%) (Figure 2E).

#### 3.4.3. Prevention of Carcinoid Crisis with Octreotide

All studies used a prophylactic octreotide strategy on at least a portion of their study population, but the strategies consisted of different combinations of preoperative and intraoperative boluses or infusions, and not all studies had a clear comparison group (control group). Table 3 provides a summary of the prophylactic strategies using octreotide in each study and their corresponding results. Appendix A Table A2 lists other octreotide uses in each study in addition to the prevention strategy, including long-acting SSA as well as treatment use for carcinoid crisis.

**Figure 3:**
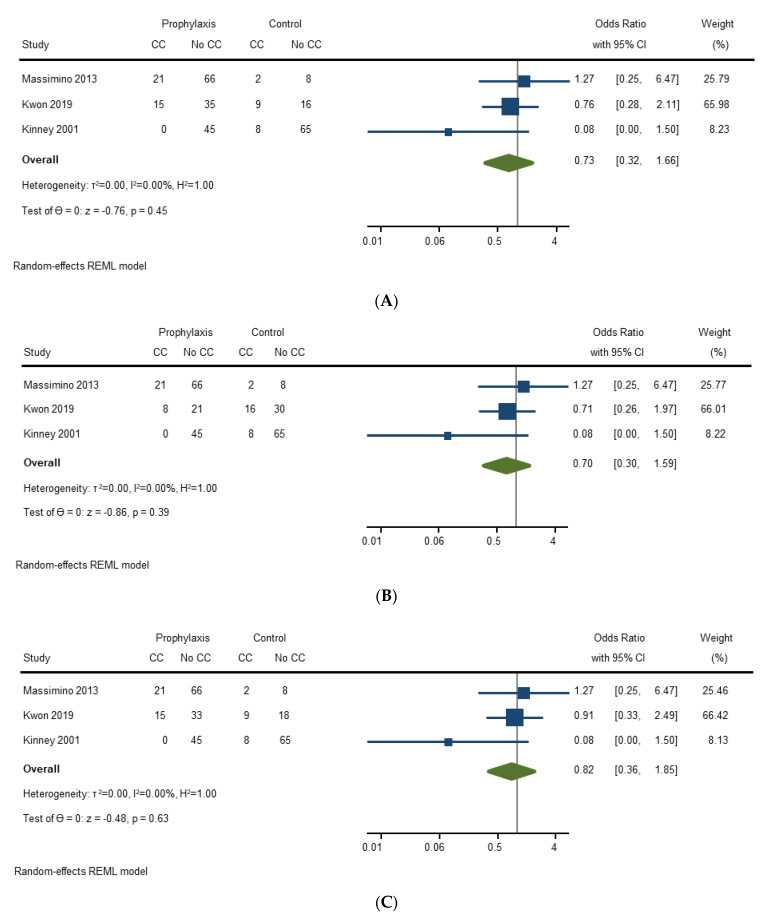
(**A**) Forest plot evaluating efficacy of octreotide in prevention of carcinoid crisis, all strategies included (pre- and intraoperative bolus/infusion); (**B**) Forest plot evaluating efficacy of octreotide in prevention of carcinoid crisis, preoperative octreotide (dominant strategy); (**C**) Forest plot evaluating efficacy of octreotide in prevention of carcinoid crisis, intraoperative octreotide (dominant strategy) [20,21,27].

Three studies (290 surgeries) assessed the effect of prophylactic octreotide on development of carcinoid crisis. Use of prophylactic octreotide compared to no prophylactic octreotide was not associated with lower pooled odds of developing carcinoid crisis [OR 0.73 (95% CI, 0.32–1.66); *p* = 0.45]. There was no heterogeneity between studies (I2 = 0%) (Figure 3A). Sensitivity analysis was conducted using each of the two dominant strategies (preoperative versus intraoperative) discretely evaluated by Kwon et al. [27], with the pooled efficacy found to remain non-significant (Figure 3B,C).

#### 3.4.4. Prognosis of Carcinoid Crisis

A total of 5 studies reported on postoperative outcomes in relation to carcinoid crisis (Table 4) [21,22,24,26,27].

**Figure 4:**
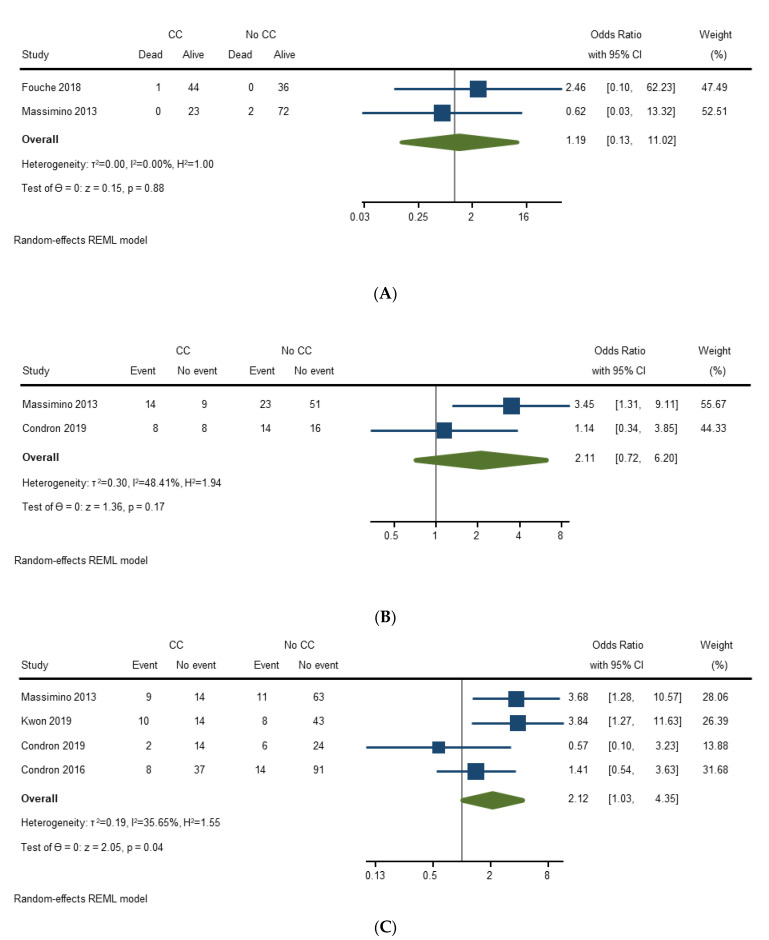
(**A**) Forest plot evaluating prognosis of carcinoid crisis—postoperative mortality; (**B**) Forest plot evaluating prognosis of carcinoid crisis—any postoperative complication; (**C**) Forest plot evaluating prognosis of carcinoid crisis—major postoperative complication [21,22,24,26,27].

Two studies (178 surgeries) assessed the effect of carcinoid crisis on postoperative mortality. Carcinoid crisis compared to no carcinoid crisis was not associated with higher pooled odds of postoperative mortality [OR 1.19 (95% CI, 0.13–11.02); *p* = 0.88]. There was no heterogeneity between studies (I2 = 0%) (Figure 4A).

Two studies (143 surgeries) assessed the effect of carcinoid crisis on having any postoperative complication. Carcinoid crisis compared to no carcinoid crisis was not associated with higher pooled odds of having any postoperative complication [OR 2.11 (95% CI, 0.72–6.20); *p* = 0.17]. There was moderate heterogeneity between studies (I2 = 48.4%) (Figure 4B).

Four studies (368 surgeries) assessed the effect of carcinoid crisis on having a major postoperative complication. In this analysis, “major” complication was defined as the highest Clavien–Dindo grade category evaluated by a study. Three studies classified Clavien–Dindo grade III and above as major complication [21,22,26] and one study classified Clavien–Dindo type II and above as major complication [27]. Carcinoid crisis compared to no carcinoid crisis was associated with higher pooled odds of having a major postoperative complication [OR 2.12 (95% CI, 1.03–4.35); *p* = 0.04]. There was moderate heterogeneity between studies (I2 = 35.7%) (Figure 4C). Sensitivity analysis was conducted by excluding a study that included patients with lung primary NETs [26], which resulted in a stronger association between carcinoid crisis and major postoperative complications [OR 2.59 (95% CI, 1.31–5.12); *p* = 0.01].

## 4. Discussion

The prevalence of patients with NETs continues to increase [28], with surgery being a critical treatment option for locoregional and/or metastatic disease [4,6,7,9]. Intraoperative carcinoid crisis, though felt to be common, has only been studied by a few groups and much of the current knowledge and/or management approach is still guided by dogma and small case reports [11,12,29,30]. We performed a systematic review and meta-analysis pooling the available data to inform critical aspects of carcinoid crisis, and specifically to examine the efficacy of prophylactic octreotide-based regimens for preventing carcinoid crisis. Eight studies met our inclusion criteria encompassing a total of 943 operations. We found carcinoid crisis to be common, occurring in 1 in 5 patients (incidence = 19%) in the pooled data. The risk of carcinoid crisis was increased in patients with liver metastases and decreased in male patients. Other characteristics that were traditionally thought to be risk factors, including carcinoid syndrome and carcinoid heart disease, were not significantly associated with an increased risk of carcinoid crisis. Multiple prophylactic regimens using octreotide were identified, with varying doses and strategies (routes and timing) for administering octreotide, with no standardized approach tested across studies. Despite this variability, none of the individual or pooled data showed a significant decrease in carcinoid crisis with use of prophylactic octreotide. Lastly, patients with carcinoid crisis had a higher risk of developing major postoperative complications. The findings of this systematic review are noteworthy as they clarify important aspects related to the incidence, risks, prognosis, and prevention of carcinoid crisis, while also bringing to light important challenges with existing data—specifically related to lack of standardization in terms and treatment approaches, guiding focused efforts and future needs.

This analysis included studies with surgical patients that had midgut NETs and/or those with neuroendocrine liver metastasis. As expected, data published on intraoperative carcinoid crisis was also primarily available for patients with midgut NET and NET liver metastasis from other sites—including lung and pancreas, representing the high-risk population. The incidence of carcinoid crisis ranged from 0 to 56%, depending on the study. This wide range may be due to differences in study populations; in 5 studies, having metastatic disease was part of the inclusion criteria, while other studies included only patients with small bowel primary tumors, and others included patients with other primary tumor locations, such as the pancreas or lungs. Additionally, though all studies included surgical patients, the types of procedures performed varied. Differences in the definition were primarily based on primary signs, duration of signs, and assignment method (i.e., requirement by anesthesiologist/surgeon—ruled out other causes). As expected, studies that included a minimum time requirement to define carcinoid crisis had a lower pooled incidence (10%) than studies that did not have a time requirement to define carcinoid crisis (31%). Fouché et al. had the broadest outcome definition by including probable events and had the highest incidence (56%) [24]. Our findings further support the published findings that carcinoid crisis is a common occurrence, and that CC definition should be guided by the presence of hemodynamic changes not otherwise explained by surgical or anesthetic treatments. Other cited signs and symptoms can help further characterize the event.

When examining populations at risk of carcinoid crisis, our results support the long-standing observation that patients with liver metastases are at highest risk of experiencing intraoperative carcinoid crisis [2]. Interestingly, other characteristics thought to be associated with intraoperative carcinoid crisis from previous reports [10,13,31] were not found to be associated with an increased risk of carcinoid crisis, specifically carcinoid syndrome and carcinoid heart disease. These results may be related to potential misclassification bias, as none of the studies focused on collecting and confirming occurrence of these risk factors in a standardized manner. However, these findings may also be explained by a difference in pathophysiology pathways for each of these two different syndromes. Notably, though carcinoid crisis is typically associated with carcinoid tumors, especially small bowel tumors, we found that patients with NETs and liver metastases from other primary tumor locations, such as the pancreas, can present with carcinoid crisis. Future studies aimed to examine features of carcinoid crisis and prevention strategies, should include all patients at risk, including those with NET liver metastasis and those with GEP tumors with or without liver metastases.

The primary goal of this study was to examine the efficacy of prophylactic octreotide for preventing carcinoid crisis. Four studies could not be included in the pooled analysis because there was no control group for comparison with all patients intended to receive prophylactic octreotide [22,23,24,26], and a 5th study was excluded as it was not focused on the prevention of carcinoid crisis [25]. Importantly, 2 of these studies [22,26] compared their data to historic controls treated within the same setting and standards, with the consistent observation of no benefit of octreotide use. Three studies were included in the pooled analysis; with different strategies for octreotide administration, that varied by timing (pre- and intra-operative) and dose (bolus ranging from 50 to 1100 µg, and infusions ranging from 50 to 300µg/hour) between studies [20,21,27]. Kwon et al. examined the use of preoperative octreotide or intraoperative octreotide use, and a pooled analysis or both strategies (preoperative and intraoperative) on preventing carcinoid crisis [27]. We conducted a pooled analysis using all strategies from the 3 studies and performed sensitivity analysis by pooling only the studies with the preoperative octreotide as the dominant strategy and with intraoperative octreotide as the dominant strategy. None of the pooled results were significant. These findings provide additional evidence supporting the concept that use of prophylactic octreotide may not prevent carcinoid crisis and hence its routine use must be further studied and reconsidered. A recent study by Wonn et. al. [32] from Oregon Health and Science University examined their group’s transition to a “no-perioperative octreotide” approach in 171 patients and the authors reported an incidence of carcinoid crisis of 25%—within the same range when compared to their previous approaches using octreotide prophylaxis [22,26]. Future efforts should focus on alternative protocols for carcinoid crisis prevention, including multi-drug regimens and/or other “targeted” therapies; prior studies have shown an association between preoperative serum serotonin levels before incision and carcinoid crisis, which has led to investigators considering preventive strategies with serotonin synthesis inhibitors (i.e., preoperative telotristat ethyl) [11,32,33]. Most importantly, these efforts should be led within the framework of a randomized trial, including patients at risk, and following a standardized approach that can be appropriately examined and systematically replicated if successful.

Despite differences in the NET studies, we were able to conduct a meta-analysis to assess the impact and clinical significance of a carcinoid crisis event. Our findings showed that though carcinoid crisis was not associated with higher odds of postoperative mortality, it was associated with higher odds of major postoperative complications. One study examined specific postoperative complications and found that carcinoid crisis patients had higher risk of postoperative pulmonary embolism and tachyarrhythmia requiring nodal blockers [27]. However, these results could not be pooled with any other studies. Several studies also identified potential risk factors that could not be included in the pooled analysis but may be worth considering in future studies including preoperative hypertension [23], use of epidural catheter [21], estimated blood loss [22], duration of anesthesia [22], and pre-incision serotonin [26]. Although these findings are limited by the heterogeneity of the populations included, these findings have important implications in selecting patients in future studies.

This study has several limitations, including the non-randomized nature of the included studies and potential for publication bias. Similarly, it should be noted that our primary outcome (efficacy of octreotide on preventing carcinoid crisis) was ultimately derived from only 3 studies appropriately addressing this question, with the need for additional well-conducted trials to further study this association. However, the quality of the studies and risk of bias were assessed with a validated approach and revealed an overall fair quality of the studies. Further, it is possible that our exclusion criteria may have omitted important results, although this appears unlikely as the drivers for exclusion were primarily based on relevance to the primary question and sample size of the studies. This meta-analysis included a study with lung NETs with liver metastasis [26]. Existing studies have found that lung NETs may have distinct characteristics compared to GEP NETs [34,35]. To further assess this, sensitivity analysis was conducted excluding the study, which did not affect the major findings of this meta-analysis. Lastly, the findings may be limited by the variability in the preventive strategies used, including short-acting octreotide regimens for prevention and long-acting SSA for symptom and/or tumor control, yet we attempt to account for these limitations by conducting sensitivity analyses and reporting on the heterogeneity of the population. Further, these findings emphasize an important gap in the field and guides efforts for future research approaches.

## 5. Conclusions

In summary, this meta-analysis found that carcinoid crisis is common in patients with midgut neuroendocrine tumors—occurring in 1 in 5 patients having abdominal operations, and that the risk is particularly increased for those with liver metastasis. Similarly, we found evidence regarding the questionable role of prophylactic octreotide for preventing carcinoid crisis, and the need for future appropriately designed studies to better define the role of perioperative octreotide in this population. These findings contribute as a first step to evidence-based practices as opposed to dogma-driven approaches to managing NET patients during surgery. Given the high incidence of carcinoid crisis and its clinical implications, future efforts to identify preventive and management strategies for carcinoid crisis should continue. Findings from this study emphasize the importance to frame these efforts in the context of a standardized definition for carcinoid crisis and a systematic approach for prevention.

## Figures and Tables

**Table 1:** Study characteristics, definition, and incidence of carcinoid crisis (CC) across included studies.

Study	Study Design	Study Population	N *	Definition of CC	Incidence of CC	Comments
Kinney (2001) [20]	Retrospective Single institution January 1983–December 1996	**Tumor Type:** Metastatic carcinoid tumors **Carcinoid Syndrome:** Not specified, patients with preoperative carcinoid syndrome symptoms included **Carcinoid Heart Disease:** 20.2% (24/119) **Surgical Procedures:** Hepatic arterial ligation, resection or biopsy of hepatic metastases, hepatic carcinoid cryotherapy, and small or large bowel resection or diversion—alone or combined	119	Flushing, urticaria, ventricular fibrillation, SBP < 80 mmHg for >10 min, bronchospasm, acidosis (pH < 7.2), tachycardia (pulse > 120 bpm).	6.7% (8/119)	8 patients had intraoperative complication. 15 patients had perioperative complication or postoperative death.
Massimino (2013) [21]	Retrospective Single institution January 2007–January 2011	**Tumor Type:** Gastrointestinal carcinoid tumors, metastases included **Carcinoid Syndrome:** 58.8% (57/97) **Carcinoid Heart Disease:** 2.1% (2/97) **Surgical Procedures:** Abdominal operations including hepatic resection, bowel resection, cholecystectomy, resection of mesenteric mass, and others	97	SBP ≤ 80 mmHg for ≥10 min, report of hemodynamic instability (hypotension, sustained hypertension, or tachycardia) not due to acute blood loss or other obvious causes, or if anesthesiologist or attending surgeon declared carcinoid crisis occurred in anesthesia record or operative report.	24% (23/97)	18 patients had prolonged hypotension. 5 patients had hemodynamic instability consistent with carcinoid crisis.
Condron (2016) [22]	Prospective Single institution January 2011–August 2014	**Tumor Type:** Carcinoid tumors, metastases included **Carcinoid Syndrome:** 74% (111/150) **Carcinoid Heart Disease:** 3% (5/150) **Surgical Procedures:** Bowel resection, hepatic resection, resection of mesenteric nodal mass, and others	150 (127 patients)	SBP < 80 or > 180 mmHg, heart rate > 120 beats per minute, or if patient displayed physiology that would be expected to cause end organ dysfunction if sustained. Not attributable to other causes. Consensus of the surgeon and attending anesthesiologist necessary to declare crisis.	30% (45/150)	-
Woltering (2016) [23]	Retrospective Single institution May 2006–March 2012	**Tumor Type:** Small bowel neuroendocrine tumors with distant metastases **Carcinoid Syndrome:** 85.2% (150/176) “potential” carcinoid syndrome **Carcinoid Heart Disease:** N/A **Surgical Procedures:** Not specified	179 (150 patients)	SBP < 80 mmHg for >10 min that could not be explained by other factors. Anesthesia or surgical record noting intraoperative hemodynamic instability (hypertension, hypotension, or tachycardia) or containing the word “crisis” was further reviewed for possible carcinoid crisis.	3.4% (6/179)	Operations described as “cytoreductive surgeries.” No additional details.
Fouché (2018) [24]	Retrospective Single institution January 2007–December 2015	**Tumor Type:** Small bowel neuroendocrine tumors, metastases included **Carcinoid Syndrome:** 60.4% (49/81) **Carcinoid Heart Disease:** 8.6% (7/81) **Surgical Procedures:** Small bowel neuroendocrine tumor resections (operations for other neuroendocrine tumor location or for hepatic metastases only were excluded); 12/81 had liver resection	81	**Highly probable intraoperative carcinoid syndrome (ioCS):** Rapid (≤ 5 min) heart rate or arterial blood pressure changes ≥40%, not explained by surgical/anesthetic management and regressive ≥20% after octreotide bolus injection. **Probable ioCS:** Did not meet all criteria of highly probable ioCS. **Suspected ioCS:** Anesthesia record has octreotide injection due to manifestation that did not meet criteria for highly probable or probable ioCS. **Carcinoid crisis:** Life-threatening ioCS refractory to octreotide boluses.	ioCS: 55.6% (45/81) CC: 0% (0/81)	Main outcome is intraoperative carcinoid syndrome (ioCS). Multiple instances of ioCS recorded (139 instances for 45 patients). Authors note octreotide protocol was respected for 64 patients; 11 patients had lower doses.
Kinney (2018) [25]	Retrospective Single institution January 1997–June 2015	**Tumor Type:** Neuroendocrine tumors with liver metastases **Carcinoid Syndrome:** Yes (no % given) **Carcinoid Heart Disease:** 8.3% (14/169) **Surgical Procedures:** Partial hepatectomy (major and minor)/ablation, +/−small bowel resection, +/−hepatic artery ligation	196 (169 patients)	Sudden onset of at least 2: Flushing or urticaria not explained by an allergic reaction, bronchospasm or bronchodilator administration, SBP < 80 mmHg for >10 min not explained by volume status or hemorrhage and treated with pressors, dysrhythmia not explained by volume status or hemorrhage, or pulse > 120 bpm.	0% (0/196)	26 patients did not qualify as having a carcinoid crisis because they experienced only 1 of the criteria. Tachycardia was the most common criteria met.
Condron (2018) [26]	Prospective Single institution 2015–2017	**Tumor Type:** Small bowel or lung carcinoid tumor with liver metastases **Carcinoid Syndrome:** 65.2% (30/46) **Carcinoid Heart Disease:** Excluded **Surgical Procedures:** Elective abdominal operations including hepatic debulking, prophylactic cholecystectomy, resection of primary tumor, resection of mesenteric nodal mass	46	SBP < 80 or > 180 mmHg, pulse > 120 bpm, or if patient displayed physiology expected to cause end organ dysfunction if sustained. Not attributable to other causes. Consensus of the surgeon and attending anesthesiologist necessary.	35% (16/46)	-
Kwon (2019) [27]	Retrospective Single institution June 2012–December 2016	**Tumor Type:** Neuroendocrine tumors with liver metastases **Carcinoid Syndrome:** 46.7% (35/75) **Carcinoid Heart Disease:** 10.7% (8/75) **Surgical Procedures:** Liver resection, other (thermal ablation, bland hepatic artery embolization, chemoembolization, Yttrium-90 radioembolization)	75	Carcinoid crisis (CC): Documentation of CC by any treating physician. Hemodynamic instability (HDI): >10 min of SBP < 80, > 180 mmHg, or pulse > 120 bpm not attributed to blood loss or other causes.	32% (24/75)	1 patient had CC alone. 21 patients had HDI alone. 2 patients had both CC and HDI.

* Number of operations.

**Table 2:** Risk factors of carcinoid crisis.

Study	Risk Factors Evaluated	Risk Factors Associated
Massimino (2013) [21]	**Unadjusted:** Carcinoid syndrome, epidural catheter, epidural infusion, induction agents (propofol, etomidate, thiopental), hepatic metastases, hepatic resection, outpatient octreotide **Adjusted:** Age, gender, hepatic metastases, hepatic resection, epidural catheter	**Unadjusted:** Hepatic metastases (*p* ≤ 0.01), hepatic resection (*p* = 0.03), epidural catheter (*p* = 0.04) **Adjusted:** Hepatic metastases (*p*-value not specified) *
Condron (2016) [22]	**Unadjusted:** Age, estimated blood loss (EBL), duration of anesthesia, sex, carcinoid syndrome, carcinoid heart disease, location of primary tumor, hepatic metastases, mesenteric metastases, peritoneal metastases, ASA **Adjusted:** Age, ASA-3, ASA-4, carcinoid syndrome, carcinoid heart disease, duration of anesthesia, sex, hepatic metastases, mesenteric metastases, other metastases, primary tumor location, EBL	**Unadjusted:** EBL (*p* = 0.005), duration of anesthesia (*p* = 0.001), carcinoid syndrome (*p* = 0.006), hepatic metastases (*p* = 0.02) **Adjusted:** Age (*p* = 0.045), carcinoid syndrome (*p* = 0.014), duration of anesthesia (*p* = 0.022), hepatic metastases (*p* = 0.037)
Woltering (2016) [23]	**Unadjusted:** Hypertension, heart condition, potential carcinoid syndrome, preoperative SSA therapy	**Unadjusted:** Hypertension (CC 100% vs. no CC 55%) **
Fouché (2018) [24]	**Unadjusted:** Carcinoid syndrome, preoperative diarrhea, preoperative cutaneous flush, carcinoid heart disease, hepatic metastases, elevated preoperative output of 5-hydroxylindoleacetic acid, premedication with antihistamine, intraoperative vasopressor use (ephedrine, phenylephrine, noradrenaline), hepatic resection **Adjusted:** N/A	**Unadjusted:** None significant
Condron (2018) [26]	**Unadjusted:** Age, sex, carcinoid syndrome, operative procedures, resection of mesenteric nodal mass, volume of hepatic metastases, estimated volume debulked, duration of anesthesia, estimated blood loss, preincision hemodynamics, preincision serotonin, preincision histamine, preincision kallikrein, preincision bradykinin, dose of octreotide LAR at time of operation, duration of long-acting SSA treatment prior to operation **Adjusted:** Age, anesthesia time, estimated blood loss, preincision serotonin, preincision histamine, preincision kallikrein, preincision bradykinin	**Unadjusted:** Preincision serotonin (*p* = 0.0064) **Adjusted:** Preincision serotonin (OR 1.1 [95% CI 1.01–1.19], *p* = 0.015)
Kwon (2019) [27]	**Unadjusted and adjusted:** Age, sex, Eastern Cooperative Oncology Group Performance Status (ECOG), primary tumor location, tumor grade, extent of hepatic involvement by metastases, history of carcinoid syndrome, CGA greater than 2 x upper limit of normal and Urine 5-HIAA greater than 2 x upper limit of normal, long-acting SSA use in prior month	**Unadjusted and adjusted:** None significant ***

* Presence of hepatic metastases was perfect predictor, after removing hepatic metastases from the model, no other variables were significant. ** No statistical tests. *** No clinicopathologic or procedural factors were associated with CC/HDI.

**Table 3:** Prevention of carcinoid crisis with octreotide.

Study	Prophylactic Octreotide	Prophylactic Octreotide Strategy and % Patients	Other Octreotide Use	Risk Reduction (Y/N) and Strength of Association	Comments
Kinney (2001) [20]	Yes	Preoperative Bolus: 26% (31/119), median 300 μg, (range 50–1000 μg)	Intraoperative Bolus: 38% (45/119), median 350μg (range 30–4000 μg). Unclear if intraoperative bolus was administered as part of prevention technique or as treatment.	Intraoperative octreotide: Yes (*p* = 0.023)	FDA approved octreotide in 1988. Analysis on 1988–1996 data only (not all patients included), and intraoperative octreotide reduced risk (*p* = 0.010). No predictors of efficacy.
Massimino (2013) [21]	Yes	Preoperative Bolus: 90% (87/97), median 500 μg (range 100–1100 μg) Intraoperative Infusion: 8% (8/97), dose unspecified	Intraoperative Bolus: 52% (50/97), median 350 μg (range 100–5500 μg). Intraoperative bolus available as therapy.	Preoperative bolus: No (*p* = 0.77)	No predictors of efficacy.
Condron (2016) [22]	Yes	Preoperative Bolus: 100% (150/150), 500 μg Intraoperative Infusion: 100% (150/150), 500 μg/h 86% (129/150) compliance of octreotide protocol	Intraoperative Bolus: % not specified Intraoperative bolus described as part of crisis management.		100% of patients received prophylactic octreotide—no comparison group.
Woltering (2016) [23]	Yes	Preoperative Bolus: 100% (179/179), 500 μg Preoperative, intraoperative, and postoperative infusion: 100% (179/179), 500 μg/h	Intraoperative boluses are kept on hand and administered as necessary. Unclear what would trigger administration.		100% of patients received prophylactic octreotide—no comparison group.
Fouché (2018) [24]	Yes	40 μg/h (80 μg/h if prior carcinoid syndrome, hepatic metastases, or carcinoid heart disease) infusion 12–48 h prior to operation. Same dose continued for intraoperative infusion. 79% (64/81) compliance of octreotide protocol	Intraoperative Bolus: 0.5–2 μg/kg (if patient has ioCS). Intraoperative boluses were administered to treat IoCS.		Octreotide protocol was respected in 64 patients and 11 patients had lower doses. No details about octreotide administration for remaining 6 patients. No clear control group.
Kinney (2018) [25]	Yes	Preoperative Bolus: 77% (130/169), 500 μg	Intraoperative Bolus: 23% (39/169), median 500 μg (IQR 250, 650). Unclear if intraoperative bolus was administered as part of prevention technique or as treatment.	Did not evaluate efficacy of prophylactic octreotide	The clinical availability and use of SA and LAR octreotide changed over duration of study.
Condron (2018) [26]	Yes	Preoperative Bolus: 100% (46/46), 500 μg Intraoperative Infusion: 100% (46/46), 500 μg/h	None.		100% of patients received prophylactic octreotide—no comparison group. No predictors of efficacy.
Kwon (2019) [27]	Yes	Preoperative Bolus: 28% (21/75), median 150 μg (range 100–300 μg) Preoperative Infusion: 36% (27/75), median 150 μg/h (range 50–300 μg/h) Intraoperative Infusion: 64% (48/75), median 150 μg/h (range 50–300 μg/h)	Intraoperative Bolus: 27% (20/75), median 150 μg (range 20–510 μg). Unclear if intraoperative bolus was administered as part of prevention technique or as treatment.	Preoperative octreotide: No (*p* = 0.52) Intraoperative octreotide: No (*p* = 0.85) Preoperative or Intraoperative octreotide: No (*p* = 0.60)	No predictors of efficacy.

**Table 4:** Prognosis of carcinoid crisis.

Study	Mortality Rate	Post-Operative Complication Rate	Incomplete Operation/Aborted Procedure Rate	Average Length of Stay	Comments
Massimino (2013) [21]	**Unadjusted:** CC 0/23 vs. no CC 2/74	**Unadjusted:** Any complication: CC 60.9% vs. No CC 31.1% (*p* = 0.01) Major complication: CC 39.1% vs. No CC 14.9%	N/A	N/A	Postoperative period = 30 days Also significant different between minor and major complications for CC and no CC (*p* = 0.02) Major complication = Dindo Grade III+
Condron (2016) [22]	N/A	**Unadjusted:** Major complication: *p* = 0.481 **Adjusted:** Major complication: OR 0.94 [95% CI 0.23–3.67] *p* = 0.93	**Unadjusted:** CC 3/45 vs. no CC 0/105	N/A	Postoperative period length not specified Major complication = Dindo Grade III+
Fouché (2018) [24]	**Unadjusted:** CC 1/45 vs. no CC 0/36 **Adjusted:** N/A	N/A	N/A	N/A	Postoperative period length not specified No statistical analysis
Condron (2018) [26]	N/A	**Unadjusted:** Any: CC 50% vs. No CC 46.7% (*p* = 0.829) Clavien–Dindo I-II: CC 37.5% vs. No CC 26.7% (*p* = 0.447) Clavien–Dindo III-IV: CC 12.5% vs. No CC 20% (*p* = 0.523)	N/A	**Unadjusted:** Mean LOS: CC 11.6 vs. no CC 8.3 (*p* = 0.315)	Postoperative period length not specified
Kwon (2019) [27]	N/A	**Unadjusted:** Clavien–Dindo II-IV: CC 42% vs. No CC 16% (*p* = 0.01) Postoperative pulmonary embolism: CC 8% vs. No CC 0% (*p* = 0.04) Postoperative tachyarrhythmia requiring nodal blocker: CC 8% vs. No CC 0% (*p* = 0.04)	N/A	**Unadjusted:** Median LOS: CC 5.5 vs. no CC 4.0 (*p* = 0.13)	Postoperative period not specified Other outcomes evaluated that did not have association: hypotension requiring vasopressors, bleeding or coagulopathy requiring transfusions, hypoxemia requiring intensive care, infection, acute kidney injury, endoscopic or radiologic procedure, surgery, other

## Appendix A

**Table A1:** Risk of bias assessment.

Study	Is the Case Definition Adequate?	Representativeness of the Cases	Selection of Controls	Definition of Controls	Comparability of Cases and Controls on the Basis of the Design or Analysis	Ascertainment of Exposure	Same Method of Ascertainment for Cases and Controls	Non-Response Rate
Kinney (2001) [20]	Low Risk	Low Risk	Low Risk	Unclear	Unclear/High Risk	Low Risk	Low Risk	Low Risk
Massimino (2013) [21]	Low Risk	Unclear/High Risk	Low Risk	Low Risk	Low risk	Low Risk	Low Risk	Low Risk
Condron (2016) [22]	Low Risk	Unclear/High Risk	Low Risk	Low Risk	Low Risk	Low Risk	Low Risk	Low Risk
Woltering (2016) [23]	Low Risk	Unclear/High Risk	Low Risk	Low Risk	Low Risk	Low Risk	Low Risk	Low Risk
Fouche (2018) [24]	Unclear/High Risk	Low Risk	Low Risk	Unclear/High Risk	Unclear/High Risk	Low Risk	Low Risk	Low Risk
Kinney (2018) [25]	Low Risk	Low Risk	Low Risk	Low Risk	Low Risk	Low Risk	Low Risk	Low Risk
Condron (2018) [26]	Low Risk	Unclear/High Risk	Low Risk	Low Risk	Low Risk	Low Risk	Low Risk	Low Risk
Kwon (2019) [27]	Low Risk	Low Risk	Low Risk	Low Risk	Low Risk	Low Risk	Low Risk	Low Risk

**Table A2:** Summary of octreotide use across studies. Includes use of long-acting somatostatin receptor analogues, short-acting octreotide prevention, and treatment regimens (dose and route).

Study	Long-Acting Release (LAR) Octreotide			Preoperative Bolus of Octreotide		Preoperative Infusion of Octreotide			Intraoperative Bolus of Octreotide		Intraoperative Infusion of Octreotide		Comments
	% (n/d) Patients	Dose	Duration	% (n/d) Patients	Dose	% (n/d) Patients	Infusion Rate	Duration	% (n/d) Patients	Dose	% (n/d) Patients	Infusion Rate	
Kinney (2001) [20]				26% (31/119)	Median 300 μg (range 50–1000 μg)				38% (45/119)	Median 350 μg (range 30–4000 μg)			-
Massimino (2013) [21]	72% (70/97)			90% (87/97)	Median 500 μg (range 100–1100 μg)				52% (50/97)	Median 350 μg (range 100–5500 μg)	8% (8/97)		Intraoperative bolus and intraoperative infusion frequencies and percentages are calculated, not directly stated in article.
Condron (2016) [22]	Yes (% not specified)			100% (150/150)	500 μg				Yes (% not specified)		100% (150/150)	500 μg/h	86% (129/150) compliance of octreotide protocol.
Woltering (2016) [23]	83% (149/179)			100% (179/179)	500 μg	100% (179/179)	500 μg/h				100% (179/179)	500 μg/h	-
Fouché (2018) [24]						Yes (% not specified)	40 μg/h 80 μg/h if patient had prior carcinoid syndrome, hepatic metastases, or carcinoid heart disease	12–48 h	Yes (% not specified)	0.5–2 μg/kg (if patient has ioCS)	Yes (% not specified)	40 μg/h 80 μg/h if patient had prior carcinoid syndrome, hepatic metastases, or carcinoid heart disease	Difficult to understand how the hourly dose was given preoperatively. 79% (64/81) compliance of octreotide protocol.
Kinney (2018) [25]	28% (48/169)			77% (130/169)	500 μg				23% (39/169)	Median 500 μg (IQR 250, 650)			Overlapping short- and long-acting; unclear % receiving prophylaxis.
Condron (2018) [26]	100% (46/46)	30 mg (3 patients received 20 mg, 1 patient received 10 mg)	At least 28 days	100% (46/46)	500 μg						100% (46/46)	500 μg/h	
Kwon (2019) [27]	59% (44/75)			28% (21/75)	Median 150 μg (range 100–300 μg)	36% (27/75)	Median 150 μg/h (range 50–300 μg/h)		27% (20/75)	Median 150 μg (range 20–510 μg)	64% (48/75)	Median 150 μg/h (range 50–300 μg/h)	Other strategies included H-blockers.
